# An Integrated Multiomics Approach to Identify Candidate Antigens for Serodiagnosis of Human Onchocerciasis*[Fn FN2]

**DOI:** 10.1074/mcp.M115.051953

**Published:** 2015-10-15

**Authors:** Samantha N. McNulty, Bruce A. Rosa, Peter U. Fischer, Jeanne M. Rumsey, Petra Erdmann-Gilmore, Kurt C. Curtis, Sabine Specht, R. Reid Townsend, Gary J. Weil, Makedonka Mitreva

**Affiliations:** From the ‡McDonnell Genome Institute, Washington University in St Louis, Missouri 63108;; §Division of Infectious Diseases, Department of Medicine, Washington University School of Medicine, St. Louis, Missouri 63110;; ¶Division of Endocrinology, Metabolism and Lipid Research, Department of Medicine, Washington University School of Medicine, St. Louis, Missouri 63110;; ‖Department of Cell Biology and Physiology, Washington University School of Medicine, St. Louis, Missouri 63110;; **Institute for Medical Microbiology, Immunology and Parasitology, University Hospital of Bonn, Bonn, Germany 53127

## Abstract

Improved diagnostic methods are needed to support ongoing efforts to eliminate onchocerciasis (river blindness). This study used an integrated approach to identify adult female *Onchocerca volvulus* antigens that can be explored for developing serodiagnostic tests. The first step was to develop a detailed multi-omics database of all *O. volvulus* proteins deduced from the genome, gene transcription data for different stages of the parasite including eight individual female worms (providing gene expression information for 94.8% of all protein coding genes), and the adult female worm proteome (detecting 2126 proteins). Next, female worm proteins were purified with IgG antibodies from onchocerciasis patients and identified using LC-MS with a high-resolution hybrid quadrupole-time-of-flight mass spectrometer. A total of 241 immunoreactive proteins were identified among those bound by IgG from infected individuals but not IgG from uninfected controls. These included most of the major diagnostic antigens described over the past 25 years plus many new candidates. Proteins of interest were prioritized for further study based on a lack of conservation with orthologs in the human host and other helminthes, their expression pattern across the life cycle, and their consistent expression among individual female worms. Based on these criteria, we selected 33 proteins that should be carried forward for testing as serodiagnostic antigens to supplement existing diagnostic tools. These candidates, together with the extensive pan-omics dataset generated in this study are available to the community (http://nematode.net) to facilitate basic and translational research on onchocerciasis.

Onchocerciasis is a neglected tropical disease that is responsible for significant morbidity (blindness and/or severe skin disease) in sub-Saharan Africa. An estimated 37 million people in 34 countries are infected with the causative agent of onchocerciasis, *Onchocerca volvulus* (1). Large-scale public health programs based on mass treatment with the anthelmintic ivermectin have reduced *O. volvulus* infection rates in many areas (2, 3), and plans are being developed to scale up activities to eliminate the infection (4).

Improved diagnostic tools are needed for onchocerciasis elimination programs to identify hypoendemic areas that were excluded from prior control programs and to determine when transmission has been interrupted. Therefore, the lack of an ideal (*i.e.* highly sensitive and specific, point-of-care) diagnostic test for adult worms presents a significant hurdle. Identification of worm larvae (microfilariae, MF; Fig. 1) in small skin biopsies (“skin snips”) has been the standard diagnostic method for onchocerciasis for many decades. While highly specific, skin snips are insensitive in the case of light infections or infections with worms that are not reproducing (*e.g.* adolescent worms or worms that have been temporarily sterilized by drug treatments). Several PCR-based DNA detection assays and ELISA-based serodiagnostic assays have been proposed, but these are impractical for use in the field (5–9). Only one rapid-format serodiagnositic test is available at this time, the S.D. BIOLINE Onchocerciasis cassette test (the “BO TEST”) that detects IgG4 antibodies to recombinant antigen Ov16 (10, 11).

Serodiagnostic assays like the Ov16 test are particularly useful for monitoring young children toward the end of elimination programs because antibodies should be absent from individuals born after transmission has been interrupted (10). There is a long history of work to develop sensitive and specific serodiagnostic assays for onchocercerciasis (12). In the premolecular era, parasite fractions and extracts were tested for their ability to distinguish the antibody responses of infected and noninfected individuals (13–17). Later, phage display libraries were screened to identify clones expressing parasite proteins reactive with antibodies in patient sera (18). Several recombinant proteins and protein combinations were assessed (8, 9, 19–22), and the Ov16 antigen stood out due to its high specificity (6, 11, 23).

The S.D. Bioline Onchocerciasis antibody test is a promising new tool for community screening and mapping of endemic areas. However, previous studies, including studies of Ov16, have shown that patients with different disease manifestations and in different stages of infection show markedly different antibody responses (10, 24–27), which could confound even the best single-antigen serodiagnostic assay. Therefore, it would be advantageous to identify additional antigens that could work alone or in combination with Ov16 to further improve diagnostic accuracy. There have been major advances on multiple fronts since Ov16 was discovered in 1991, including methodologies and technologies for antigen identification. Therefore, the goal of this study was to use a multi-omics (genomics, transcriptomics, proteomics, and immunomics) approach to identify novel immunodiagnostic antigens that might lead to improved diagnostic tests for onchocerciasis elimination programs.

## MATERIALS AND METHODS

### 

#### 

##### Annotation of O. volvulus Gene Models

Inferred protein sequences provided with the *O. volvulus* genome assembly (WormBase WS245) were compared with known protein sequences by BLASTP (28) against the GenBank nonredundant protein database (NR, downloaded April 15, 2014) and by WU-BLAST against the following species: *Homo sapiens* (NCBI v106), *Brugia malayi* (WormBase WS238), *Loa loa* (WormBase WS238), *Wuchereria bancrofti* (Sanger v2.0), *Ancylostoma ceylanicum* (in-house assembly and annotation), *Ascaris lumbricoides* (Sanger testes v1.5), *Necator americanus* (29), *Strongyloides stercoralis* (Sanger v2.0), *Trichuris trichiura* (Sanger v2.0), *Escherichia coli* (GenBank ASM584v2), *Saccharomyces cerevisiae* (Ensembl release 24), *Candida albicans* (ASM18296v2). Putative orthologous proteins in other species were identified based on the top BLAST hit. For visualization, individual protein sequences were aligned using Clustal Omega (30), and alignments were shaded according to conservation with the *O. volvulus* putative ortholog using BoxShade (http://www.ch.embnet.org/software/BOX_form.html). Transmembrane domains and classical secretion peptides were predicted using Phobius (31, 32). Non-classical secretion signals were predicted using SecretomeP (33). Putative proteases and protease inhibitors were identified and classified using the online MEROPS peptidase database server (release 9.11) (34). Proteins were assigned to KEGG orthologous groups, pathways and pathway modules using KEGGscan (35) with KEGG release 68 (36). Associations with InterPro protein domains and Gene Ontology (GO) classifications were inferred using InterProScan (37–39). All annotations are available in supplemental Table S1.

##### RNA Isolation, cDNA Sequencing, and Gene Expression Analyses

Adult *O. volvulus* worms were isolated from nodules of onchocerciasis patients from the Ashanti region of Ghana in November 2009 and stored at −80 °C until use (40). In total, a pool of 4 male worms obtained from four different patients and 8 individual female worms obtained from 6 different patients were used for transcriptome analysis. RNA was isolated from the pooled males and the individual females using a PureLink RNA Mini Kit according to the manufacturer's suggested protocol (Ambion/Applied Biosystems, Austin, TX). RNA quality and quantity were assessed with an Agilent 2100 Bioanalyzer (Agilent Technologies, Cedar Creek, Texas) and NanoDrop ND-1000 (NanoDrop Technologies, Wilmington, DE), respectively. Total RNA was poly(A) selected using the MicroPoly(A)Purist Kit (Ambion/Applied Biosystems) according to the manufacturer's suggested protocol and reverse transcribed using the Ovation RNA-Seq V2 kit (NuGen Technologies, Inc., San Carlos, CA) with random and poly(A) primers. Paired-end cDNA libraries were generated and sequenced on the Illumina HiSeq 2000 platform according to standard protocols, and raw reads were submitted to the GenBank sequence read archive (SRA) under BioProject PRJNA219638 (supplemental Table S2). Sequence data available from the SRA for *O. volvulus* MF, L3 and adult male (accession numbers ERX200391-ERX200394, ERX200396, ERX200397) were downloaded and also employed in this study.

Relevant adapter sequences and low quality regions were trimmed, and reads were filtered based on length, complexity, and similarity to suspected contaminants as previously described (41). Remaining, high-quality reads were aligned to the *O. volvulus* genome assembly using Tophat2 (version 2.0.8, default parameters, (42)) using the genome annotation (gff3 file) as a guide. The number of reads associated with each gene was tallied using HTSeq-Count. Normalized transcript expression levels were calculated using gene lengths and read counts from HTSeq-Count output (fragments per kilobase per million reads mapped; FPKM). Genewise expression levels can be found in Table S1 for all genes detected by LC-MS.

##### Preparation of Soluble Parasite Proteome

Soluble *O. volvulus* protein extract was prepared form a pool of 3 adult female worms isolated from one patient in Bong County, Liberia in 1988 (courtesy of the late Dietrich W. Büttner) as previously described (41). Briefly, the worms were processed in a 1 ml Dounce homogenizer (GPE Scientific Limited, Leighton Buzzard, UK) in RIPA buffer (10 mm Tris-HCl, pH 7.4, 150 mm NaCl, 1% Nonidet P-40, 0.2% sodium deoxycholate, 1 mm EDTA and 10 mm NaF). The homogenate was centrifuged, and protein in the supernatant was measured using the Pierce BCA assay (Thermo Fisher Scientific, Rockford, IL).

##### Molecular Weight Fractionation of Adult Female Protein Lysate

Adult female lysate (450 μg) was separated into eight molecular weight fractions (5–150 KDa) using a GELFrEE 8100 fractionation system with an 8% cartridge (Expedeon, San Diego, CA) (43, 44). Protein fractions were precipitated using an acetone-based method and re-solubilized in 100 mm Tris-HCL pH 8.5. The protein and peptide quantities were determined using the Advanced Protein Assay kit (Cytoskeleton, Inc., Denver, CO) and a Qubit® 3.0 Fluorometer. The fractionation was also assessed using SDS-PAGE in MES running buffer (4–12% Criterion XT gels, BioRad, Hercules, CA). The highest molecular weight fraction (>120- KDa) did not contain detectable protein and was not analyzed further. The proteins in GELFrEE fractions (∼20–120 μg) were precipitated twice using 3× volumes of cold acetone.

##### Preparation of Human IgG and Immunoaffinity Purification of Adult Female Worm Proteins

A pool of 10 sera (300 μl each) from *O. volvulus* Mf and nodule positive, de-identified patients were used in immunoaffinity purification assays. Eight sera were collected in February 2014 from patients living in Lofa County Liberia, and two sera were collected from *Loa loa* and *Mansonella perstans* Mf negative patients from Kumba, Cameroon in November 1997. All onchocerciasis patient sera tested negative for circulating *W. bancrofti* antigen by the Binax ICT card test. A pool of 3 sera (1 ml each) from healthy individuals from Missouri collected in 2010 was used as negative control.

Immunoaffinity purifications were carried out as previously described (41). Briefly, total IgG was precipitated from pooled onchocerciasis patient sera and pooled control sera, respectively, and coupled to Pierce NHS-active agarose (Thermo Fisher Scientific) in spin columns (Thermo Fischer Scientific). Columns were washed, blocked, and incubated with 600 mg *O. volvulus* soluble protein overnight at 4 °C. Columns were thoroughly washed, and immune complexes were eluted with Pierce IgG elution buffer (Thermo Fisher Scientific) in 1 ml fractions. Fractions were neutralized with 1.0 m Tris pH 9.0, and aliquots were analyzed by Western blot using the original pooled patient or control sera as the primary antibody. The fraction (∼1.5 ml) with the greatest Western blot reactivity from each purification column was concentrated to ∼100 μl in a MICROCON® (YM-3) device (Millipore, Darmstadt, Germany) for protein digest as described below.

##### Protein Digestion and Peptide Purification

Protein pellets from acetone precipitation of molecular weight fractions of female worm lysate were dissolved in 20 μl of Tris buffer (100 mm, pH 8.5) containing 8 m urea. As a digestion standard, horseradish peroxidase (1 μg) was added to each sample. The proteins were reduced using TCEP (5 mm, Thermo Fisher Scientific) for 30 min, and alkylated with iodoacetamide (40 mm) (Sigma-Aldrich, St. Louis, MO) at room temperature in the dark for 30 min. The reactions were quenched with DTT (20 mm) (Sigma-Aldrich) for 15 min. The samples were digested with endoprotease Lys C (5 μg) (Sigma-Aldrich) for 30 min at 37 °C in a Barocycler (Pressure BioSciences, South Easton, MA), followed by a fourfold dilution with buffer, addition of trypsin (5 μg) and incubation at high pressure for 30 min at 37 °C. The digests were acidified with 1% TFA and the peptides were purified using SepPak cartridges and elution conditions as previously described (45). The eluted peptides were dried in a SpeedVac and dissolved in water/acetonitrile/formic acid (98%/1%/1%) and transferred to autosampler vials (SUN-SRI, Rockwood, TN) for storage at −80 °C prior to LC-MS analysis.

The immunoaffinity enriched proteins (∼ 1 ml) were concentrated in a MICROCON® (YM-3) device (Millipore) to ∼ 100 μl for filter-aided sample preparation (46). The concentrate was transferred to a MICROCON® (YM-30) device with multiple washes with Tris buffer (100 mm, pH 8.5) containing 8 m urea. After two Tris buffer exchanges (200 μl), the protein was reduced with 100 mm DTT at room temperature for 30 min and reduced with iodoacetamide (50 mm) for 30 min. The reduced and alkylated proteins were exchanged into a volatile buffer for digestion (ammonium bicarbonate, pH 7.4). Trypsin (1 μg; Cleavage after Lysine (K) or Arginine (R) except when either is followed by proline (P)) was added and digestion at 37 °C was performed overnight in a Thermomixer (Thermo Fisher Scientific). The digest was acidified to 5% formic acid. The digests were desalted using NuTips (Glygen, Columbia, MD) with sequential extraction with C_4_ and porous graphite carbon tips on a Biomek NXP robot (Beckman Coulter, Pasadena, CA). The eluates (70% acetonitrile) from the two tips were combined, concentrated to near dryness and dissolved in acetonitrile/formic acid (1%/1%) for LC-MS analysis or storage at −80 °C.

##### High-performance Liquid Chromatography with High-resolution Tandem Mass Spectrometry

A NanoLC 2D Plus System with a cHiPLC-Nanoflex and AS2 autosampler (ABSciex, Dublin, CA) was configured with two columns in parallel. One cHiPLC column (ChromXP C_18_, 200 μm × 15 cm; 3 μm particle size, 120 Å pore size) was used to inject calibrant solution (500 fmol β-galactosidase peptides in solvent A (water/acetonitrile/formic acid, 98%/1%/1%)) and the other cHiPLC column was used for sample analysis. The calibration runs were used to recalibrate the hybrid quadrupole TOF instrument every 12 h. Over the 12 h period used for spectral acquisition, the resolution and mass accuracy of the observed peptides remained >25,000 and <20 ppm, respectively (supplemental Datasets S1 and S2). The samples were loaded in a volume of 10 μl at a flow rate of 0.8 μl/min followed by gradient elution of peptides at a flow rate of 800 nL/min. The calibrant solution was eluted with the following gradient conditions with solvent B (water/formic acid/acetonitrile, 1%/1%/98%): 0, 2%; 3 min, 2%; 73 min, 50%; 83 min, 80%; 86 min, 80%; 87 min 2%; 102 min, 2%. The digests were analyzed under the following gradient conditions (time, percent solvent B): 0, 2%; 5 min, 2%; 720 min, 35%; 765 min, 80%; 770 min, 2%; 790 min, 2%.

Data acquisition was performed with a TripleTOF 5600+ mass spectrometer (AB SCIEX, Concord, ON) fitted with a PicoView Nanospray source (PV400, New Objectives, Woburn, MA) and a 10 μm Silica PicoTip emitter (New Objectives) for bottom-up proteomics. Data were acquired using an ion spray voltage of 2.9 kV, curtain gas of 20 PSI, nebulizer gas of 25 psi, and an interface heater temperature of 175 °C. The MS was operated with a resolution of greater than or equal to 25,000 (fwhm) for TOF-MS scans. For data dependent acquisition, survey scans were acquired in 250 ms from which 100 product ion scans were selected for MS2 acquisition for a dwell time of 100 ms. Precursor charge state selection was set at +2 to +5. The survey scan threshold was set to 100 counts per second. The total cycle time was fixed at 2.25 s. Four time bins were summed for each scan at a pulser frequency value of 15.4 kHz through monitoring of the 40 GHz multichannel TDC detector with four-anode/channel detection. A rolling collision energy was applied to all precursor ions for collision-induced dissociation as described in the Analyst software.

The unprocessed LC-MS data (*.wiff) were converted to *.mzML format utilizing the AB SCIEX MS Data Converter v1.3 (AB SCIEX, Foster City, CA) within PEAKS Studio, version 7.0 (Bioinformatics Solutions Inc., Waterloo, Canada) (48, 49). The resulting files were used for database searching by the PEAKS software using a single database which contained inferred proteins from *O. volvulus* (WormBase WS245), human and animal protein sequences from the UniprotKB database (*Homo sapiens* (2013), *Mus musculus* (2013), *Bos taurus* (2013), *Canis familiaris* (2013), *Oryctolagus cuniculus* (2014)), and common contaminant proteins compiled in the cRAP database (www.thegpm.org/cRAP/index.html; Retrieved 2012). A total of 235,479 entries were searched. The searches were performed with the following constraints: (1) parent ion tolerance of 25 ppm; (2) peptide fragment ion tolerance of 100 ppm (the larger error allowed for MS2 fragment identifications was used to capture lower intensity fragment ions that may have fewer detector events for accurate determination of the center of the mass measurement); (3) trypsin cleavage specificity with up to three missed cleavages and a single semitryptic peptide per sequence entry; (4) variable oxidized modification of Met and constant modification of Cys (carbamidomethylaton). Quality peptide spectral matches with the MS2 high resolution scans were determined with a false discovery rate threshold of 1% using a decoy fusion database algorithm (47). Identifications were made with <0.1% False Discovery Rate (FDR) at the protein level. Individual spectra required a minimum PEAKS score of 20 to be accepted, according to the software manual recommendations. Protein identification required at least two unique peptides sequences, not considering modifications or isobaric sequences. The inferred proteins, their quantification, and their protein group accessions for the worm proteomic study with molecular weight fractionation and the immunoaffinity enrichment study are provided in supplemental Data sets S1 and S2, respectively. The index scan numbers for viewing the MS2 spectra is provided in these tables, along with accession numbers for the proteins. Single peptide and PMF data are deposited in PeptideAtlas (PASS00679), and the complete protein list (along with the number of peptides assigned to each detected protein) are in supplemental Table S1.

##### Experimental Design and Statistical Rationale

All life cycle stages analyzed by RNAseq were represented by at least two biological replicates (8 individual adult females, 3 for L3, 2 for adult males, and 2 for MF). This allowed for the confirmation of consistency of expression and to measure variability in expression. Statistical analysis of differential expression at the RNA level was calculated using standard settings in DESeq2 (48) considering all available biological replicates.

Whole-worm lysate was separated into 8 molecular weight fractions (5–150 kDa) for proteomics analysis in order to better capture the overall proteome. The immunoaffinity purified proteomics samples included an *O. volvulus*-infected sample as well as a noninfected sample to serve as a control for background protein detection, and the whole-worm lysate additionally served as a positive control to identify likely worm-derived proteins. The samples analyzed in our proteomic studies were single-replicate; however, this data was not used to infer statistical differences in protein abundance, rather to identify presence or absence of the proteins in the different samples.

Significant enrichment and depletion of deduced proteins with various properties among protein sets was tested using a cumulative nonparametric binomial distribution test (MS Excel version 2011); property occurrence rates in the whole genome were used as the background set in all cases. FDR correction of *p* values (minimum threshold 0.05) was used to correct for multiple testing, in cases where multiple tests were ran for a single enrichment test (49).

##### Ethics Statement

All worm specimens were untreated worms collected during a chemotherapy trial for which proper IRB approval was available. We have no information linking the worms to individual patients. The use of de-identified patient sera for the development of new diagnostics was approved by the Washington University School of Medicine IRB under the protocol number 201102546.

## RESULTS AND DISCUSSION

### 

#### 

##### Parasite Material

**Fig. 1.**
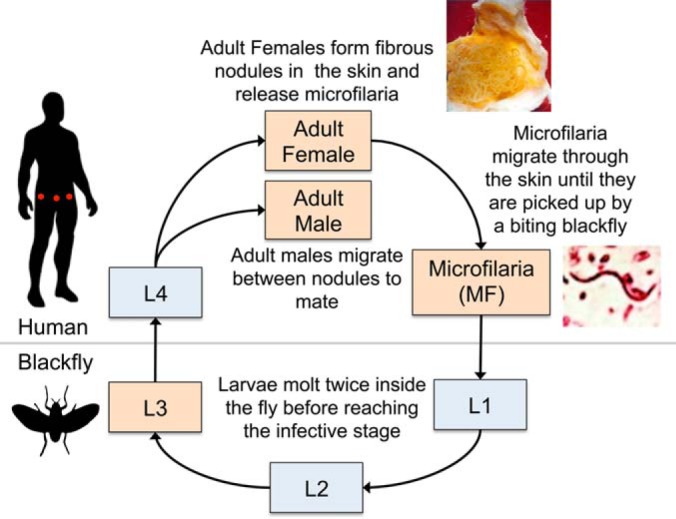
**The *O. volvulus* life cycle.** Infectious *O. volvulus* third stage larvae (L3) are transmitted to the human host by the bite of an infected blackfly (*Simulium* sp.). Over the course of a few months to a year, the worms molt (shed the outer cuticle) twice and develop into sexually mature adults. In cooperation with the host immune system, the female worms form fibrous nodules in the skin (indicated with red dots). The females remain sessile inside these nodules indefinitely whereas adult males migrate between the nodules to mate. Patent females can release thousands of microfilarial offspring per day, which migrate through the skin until they are picked up by a biting blackfly. The larvae molt twice inside the fly before reaching the infective stage. Light-orange boxes indicate stages for which RNAseq data was available and used in the current study.

This study placed a particular emphasis on adult female worms for several technical and biological reasons. Technically, the adult females are large (∼40 cm) enough to yield sufficient material for RNAseq and proteomic analyses, and relatively easy to isolate compared with other stages because they reside in subcutaneous nodules (Fig. 1). Second, they are responsible for a significant fraction of worm excretory/secretory products, presumably due to the process of birthing offspring (50), which may trigger an antibody response from the host. Third, they contain developing offspring, so microfilarial antigens will be represented in adult female RNA and lysate to some extent.

##### Annotation and Conservation of O. volvulus Predicted Proteins

**Fig. 2.**
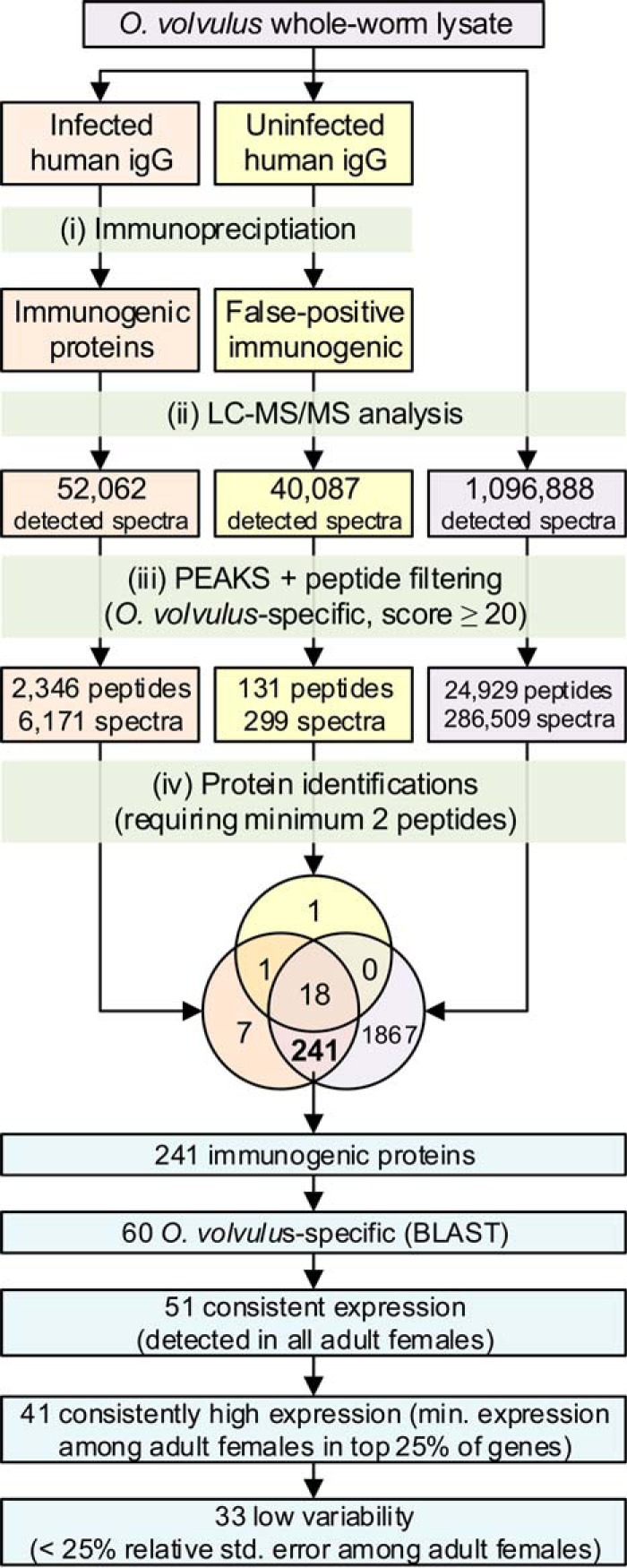
**Flowchart description of the experimental procedure and computational analysis of candidate serodiagnostic proteins.**

The identification of proteins by mass spectrometry relies on a sequence database searching approach wherein acquired MS2 spectra are matched to database peptide sequences after *in silico* endoprotease digestion (Fig. 2). The predicted protein sequences from an unpublished draft version of the *O. volvulus* genome (WormBase WS245) were used for our analyses. Functional annotations were inferred based on sequence similarity (supplemental Table S1).

Primary sequence similarity searches were used to determine the level of conservation shared between proteins from *O. volvulus* and relevant species (*i.e.* filarial nematodes and soil-transmitted nematodes with overlapping geographical distributions, the human host, and representative bacterial and yeast species; *see* Methods). This more targeted approach was preferred over searches against NR because NR contains closely related species that are not pertinent to our search for an appropriate serodiagnostic antigen (*e.g. Onchocerca* parasites of cattle). In total, 4753 inferred proteins were considered *O. volvulus*-specific in this analysis using a cutoff of 70% sequence identity over 70% sequence length (supplemental Fig. S1, supplemental Table S1).

##### RNA-level Expression of Genes Encoding O. volvulus Proteins

An RNAseq approach was used to assess the expression levels of predicted *O. volvulus* genes among adult worms (8 females and a pool of males). Comparison of global gene expression among these samples indicated a vast difference between males and females, but also a degree of diversity among the females (supplemental Fig. S2). The expanded RNAseq analysis of the eight adult female worms indicated expression signals for 11,508 of 12,143 genes (94.8%). Of those, 7361 genes had expression signals in all eight of the individual females studied (with a minimum breadth of RNAseq read coverage ≥50%); 2820 of these genes were considered to have consistently high expression (with the minimum expression level among the eight adult females being in the top 25% of all genes) and low variance (with less than 25% standard error, relative to the average expression value; supplemental Fig. S3; supplemental Table S1).

##### Proteomic Analysis of Adult Female Lysate

**Fig. 3.**
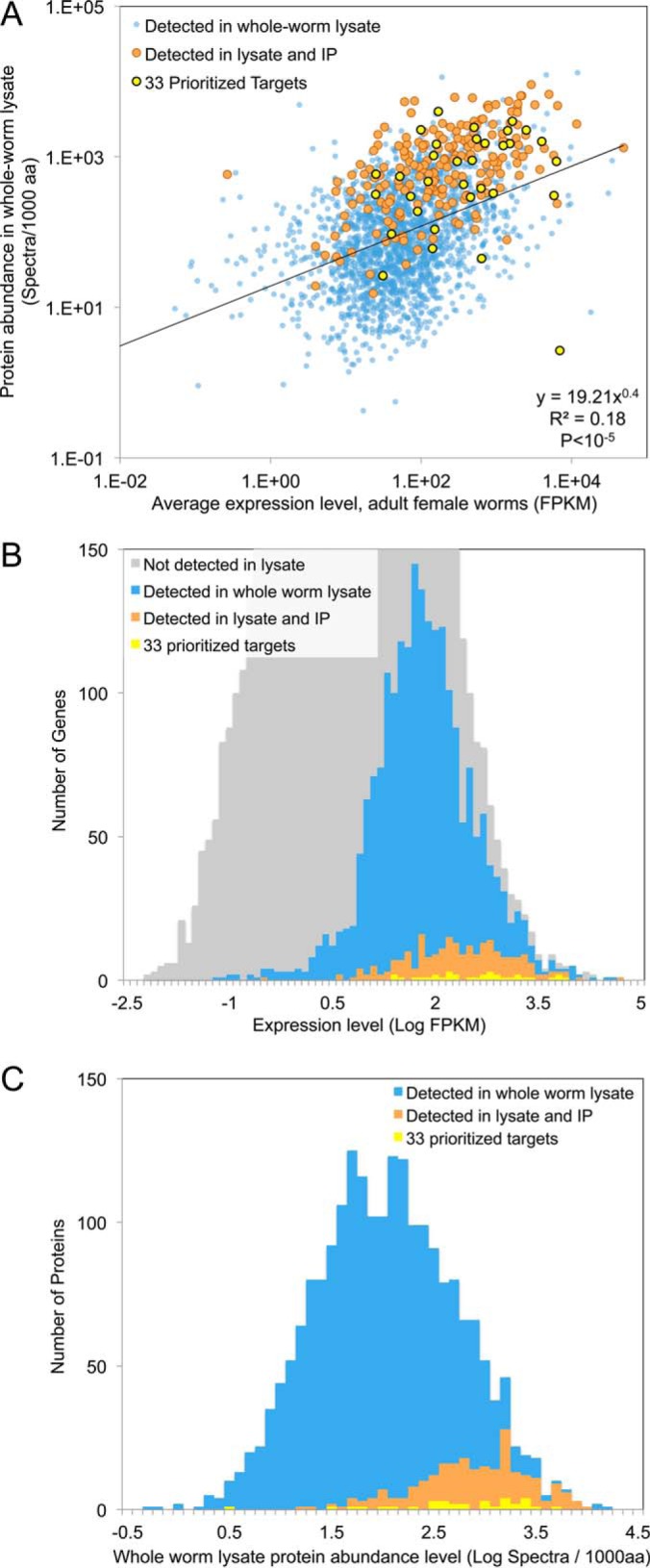
**Expression of inferred *Onchocerca volvulus* genes at the RNA and protein levels.** Gene expression and abundance levels of proteins detected in the whole-worm lysate proteomics dataset, the immunoprecipitation dataset, and the 33 prioritized serodiagnostic candidates. *A*, Protein abundance levels are significantly correlated with transcript expression levels for detected genes. *B*, Detected proteins showed high transcript expression levels. *C*, Proteins detected in the immunoprecipitation sample were among the most highly abundant proteins in the whole worm lysate.

Soluble protein from mature, adult female worms was fractionated and subjected to analysis by liquid chromatography-MS (LC-MS)^1^ in order to assess expression at the protein level. We identified 24,898 unique peptide sequences that were mapped onto 2126 *O. volvulus* proteins (supplemental Table S1), a number consistent with our expectations based on previous analyses of total parasitic worm lysates using similar methods (41, 51, 52). These proteins were enriched for signal peptides for secretion and depleted for transmembrane domains (*p* < 10^−5^ and *p* = 2 × 10^−5^, respectively). Spectral counts were used to obtain an estimate of protein abundance. As expected, there is a degree of correlation between transcript and protein expression levels (Pearson correlation = 0.20, *p* ≤ P −^5^; compared with 0.20 in the parasitic roundworm *A. suum* (53) and 0.19 in yeast (54)), and proteins detected in the worm lysate were likely to be represented at relatively high expression levels in the RNAseq datasets (Figs. 3*A* and 3*B*).

##### Identification of Immunoreactive Proteins

Immunoreactive *O. volvulus* proteins were purified from adult female lysate by immunoaffinity enrichment with IgG from human sera and identified after endopeptidase digestion and LC-MS (supplemental Table S1). A total of 248 proteins were found among those bound by pooled onchocerciasis patient IgG but not IgG from healthy North Americans (Fig. 2). All but seven of these were also found in the whole worm proteome (supplemental Table S1), and they tended to be detected at relatively high expression levels in the female worm RNAseq data and in the whole worm proteome (Figs. 3*B* and 3*C*). This approach identified many of the serodiagnostic targets that have been proposed and characterized over the past 25 years (Table I). Oddly, Ov16 and Ov33, two of the major serodiagnostic antigens for onchocerciasis, were not included in our list of high priority antigens because they were bound by control human IgG. This cross-reactivity could be due to the use of total IgG in immunoaffinity purifications rather than IgG4. Many available serodiagnostic assays, like the Ov16 rapid test and Ov33 ELISA (9, 11) measure IgG4 antibodies because this antibody subclass provides higher specificity than total IgG for helminth infections (55). Otherwise, there must be a quantitative difference in the titer of reactive IgG in infected patients compared with uninfected controls because both of these antigens have proven useful in diagnostic assays.

**Table I:** Previously described O. volvulus serodiagnostic antigens

Published name(s)	References	WS245 name	Immunoprecipitation assays		Prioritization (“Pass”, or reasons for filtering)
			Infected Human	Control Human	
Ov-RAL-2/Ov17	(18, 24)	OVOC9988	Yes	-	Pass
Ov7, Ov-CPI-1, Ov-CPI-2, OC9.3	(20, 58)	OVOC7453	Yes	-	Pass
Ov1-CF	(62)	OVOC8446	Yes	-	Too conserved
OvSOD1	(25, 63)	OVOC11517	Yes	-	Too conserved
Ov20, Ov-FAR-1, OvMBP/11, MOv2	(7, 64)	OVOC8754	Yes	-	Too conserved
Ov103, Ov-MSA-1	(19)	OVOC4230	Yes	-	Too conserved
Ov9M/Ov-CAL-1	(65)	OVOC860	Yes	-	Too conserved
Ov-FBA-1	(66)	OVOC7786	Yes	-	Too conserved
Ov-ENO	(67)	OVOC9778	Yes	-	Too conserved
Ov16	(6, 11)	OVOC12871	Yes	Yes	Recognized by control IgG
Ov33, Ov-API-1, OC3.6	(9, 59)	OVOC9984	Yes	Yes	Recognized by control IgG, too conserved
OvB20	(68, 69)	OVOC9222/5	-	-	
MOv14, OvTrop, Ov-TMY-1	(70, 71)	No match	-	-	
OvGST1	(72, 73)	OVOC7321	-	-	
M3, M4	(74)	OVOC12628	-	-	
RAL-1	(18)	OVOC7911	-	-	
Ov-ALT-1	(75)	OVOC12769	-	-	
Ov-ASP-1	(76, 77)	OVOC9575	-	-	
Ov-CHI-1, Ov-CHI-2	(78, 79)	OVOC12569	-	-	
Ov-B8	(80)	OVOC3177	-	-	
Ov-MSP2	(81, 82)	OVOC9033/4	-	-	

##### Characterization of Putative Diagnostic Antigens

**Fig. 4.**
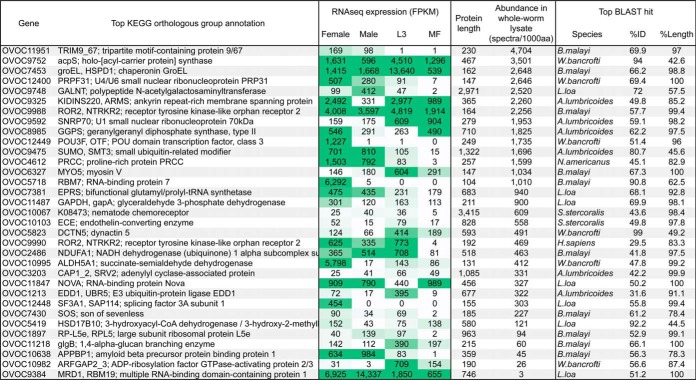
**Characterization of 33 highlighted serodiagnostic candidates.** KEGG annotations, stage-specific transcript expression levels, abundance in total worm proteome, and phylogenetic conservation of all 33 prioritized serodiagnostic candidate proteins. In applicable cases, global percent ID and percent length were summed over multiple high-scoring segment pairs.

Proteins of interest from the immunoprecipitation study (*n* = 241; Fig. 2) were further characterized based on specific properties desirable for serodiagnostic antigens as outlined in Fig. 2. Although a diagnostic test should be both sensitive and specific, specificity is particularly important for tests used in community-wide screening. Therefore, it is advantageous to select *O. volvulus* proteins that are not highly conserved with orthologs in relevant species (*i.e.* humans and other parasites that commonly infection humans in *O. volvulus* endemic areas). One hundred eighty one of the 241 immunoreactive proteins shared greater than 70% amino acid sequence identity over more than 70% of the total protein length with orthologs from relevant species. It is difficult to predict antibody cross-reactivity based on global sequence similarity, but this high level of conservation makes these proteins less attractive as candidate immunodiagnostic antigens than those that are less conserved. Of the 60 remaining protein candidates (supplemental Table S3), 51 were expressed at detectable levels in all eight of the female worms in the RNAseq arm of this study, and 33 of these showed consistently high expression among the individual worms (Fig. 4, supplementary Table S1).

##### Discussion of Select Candidate Antigens in Light of Presented Multi-omics Data

The 33 *O. volvulus*-specific and consistently expressed serodiagnostic antigens highlighted in this study are listed in Fig. 4. While several diagnostic antigens previously described in the literature were identified in the immunoprecipitation study, most of these failed in our prioritization scheme due to high levels of sequence conservation with orthologs in relevant species (*i.e.* ≥70% ID over ≥70% length), particularly *W. bancrofti* and *L. loa*, two filarial nematodes with geographical distributions that overlap that of *O. volvulus* (Table I). As previously mentioned, Ov16 and Ov33 failed our prioritization because they were present among the antigens pulled-down by control human IgG. Though Ov33 did not meet our blast specificity cutoffs, Ov16 would have qualified for our priority list if it had not been bound by control IgG. Only two previously described antigens (RAL-2 and Ov7) passed through all of our prioritization filters.

RAL-2 was originally identified as a putative vaccine candidate because it was recognized by rabbit antibodies raised against *O. volvulus* infective larvae (18). A later study showed that 88% of people with *O. volvulus* microfiladermia had antibodies reactive with RAL-2, but no specificity results were reported (24). Ov-RAL-2 shares 58% sequence identity at the amino acid level with its closest ortholog, the Bm14 antigen from *B. malayi* that has been widely used for serodiagnosis of lymphatic filariasis (56, 57). Because sera from some onchocerciasis patients contain antibodies reactive with Bm14, it is possible that *O. volvulus* RAL-2 will also have specificity issues (56). Further testing will be needed to determine if this is the case.

Like RAL-2, Ov7/OC9.3 was identified by screening a cDNA expression library with sera from infected patients and animals (20, 58). This antigen showed promising specificity, but sensitivity was in the range of 81–84% in patients with microfilaremia (20, 59, 60). Although RNAseq data indicate peak expression of this gene in the L3 stage, one study of experimentally infected chimpanzees showed that antibodies against OC9.3 were usually only detectable after the onset of microfilaremia (60); however, the kinetics of the antibody response may be different in humans.

Although the remaining 31 new candidates met our cutoffs for global sequence similarity to orthologs from relevant species, several were found to contain smaller regions of high sequence conservation upon closer inspection. For instance, portions of OVOC9752 (the candidate with second-greatest abundance in the total proteome) share very high sequence identity (95% ID over 42.6% length) with a thioredoxin peroxidase from *W. bancrofti*. Similarly, OVOC8985, contains an N-terminal region with high homology (95% ID over the first 230aa) to triosephosphate isomerases from several filarial species. These proteins may still make attractive serodiagnostic antigen candidates, as it is possible to express recombinant peptides that represent only the unique region(s) of the parent sequence. However, this should be considered with caution, as the conserved region could contain the epitope(s) that interact with patient IgG.

Among our novel candidates, OVOC4612 showed relatively high expression in the total worm proteome and relatively low sequence conservation with relevant orthologs (supplemental Fig. S4). Like the Ov16 antigen, RNAseq data indicates that peak transcription of this gene occurs in adult worms (Fig. 4). Though studies of infected chimpanzees indicated that antibodies against Ov16 were detectable prior to the appearance of MF in the skin, they still took several months to appear (61). Similar RNAseq expression profiles do not guarantee similar protein expression profiles or immune responses, but it is possible that antibodies against this protein could also take several months to become detectable considering the timing Ov16 seropositivity. In contrast, antibodies against OC3.6/Ov33 are detectable much sooner after infection compared with antibodies against Ov16 (61). RNAseq data indicates that OC3.6 shows peak expression in adult females, but it is also highly expressed in L3. OVOC2486, another novel candidate with low sequence conservation and a putative ShTK domain (supplemental Fig. S5), has an expression profile similar to OC3.6 (Fig. 4), so it is possible that antibodies against this protein could appear sooner than those targeting proteins that are not strongly expressed until later in the life cycle.

## CONCLUSIONS

This project demonstrated how a multi-omics approach can be used to efficiently identify parasite candidate antigens that may be useful for serodiagnosis. The knowledge-based prioritization scheme that we employed (limited similarity to orthologs in other parasites or in humans, expression in relevant parasite life cycle stages, and consistent expression in individual adult worms) illustrates one way to select candidates that warrant further investigation. The comprehensive database developed during this study will allow us and others in the scientific community to explore different prioritization criteria to select potential targets for diagnosis, drugs, or vaccines as they see fit. We plan to validate our prioritization schema by studying the candidate antigens' reactivity with antibodies in individual serum samples from patients with onchocerciasis and other nematode infections. Thus, future research will determine the utility of proteins identified in this study (alone or in combination with other proteins) for diagnosis of individual patients or for mapping or monitoring onchocerciasis elimination programs.

## Supplementary Material

Supplemental Data

## Footnotes

^1^ The abbreviations used are:

LC-MS: high performance nano-liquid chromatography interfaced to a high-resolution hybrid quadrupole-time-of-flight mass spectrometer
RNAseq: high throughput sequencing of mRNA
KEGG: Kyoto Encyclopedia of Genes and Genomes
NR: GenBank nonredundant protein database
FDR: false discovery rate.

## References

[1] (2010) African Programme for Onchocerciasis Control - report of the sixth meeting of National Task Forces, October 2009. Releve epidemiologique hebdomadaire / Section d'hygiene du Secretariat de la Societe des Nations = Weekly epidemiological record / Health Section of the Secretariat of the League of Nations 85, 23–28 20095110

[2] Coffeng L. E., Stolk W. A., Zoure H. G., Veerman J. L., Agblewonu K. B., Murdoch M. E., Noma M., Fobi G., Richardus J. H., Bundy D. A., Habbema D., de Vlas S. J., and Amazigo U. V. (2014) African programme for onchocerciasis control 1995–2015: updated health impact estimates based on new disability weights. PLoS Neglected Tropical Diseases 8, e2759 24901642 10.1371/journal.pntd.0002759 PMC4046979

[3] Centers for Disease, C., and Prevention (2013) Progress toward elimination of onchocerciasis in the Americas - 1993–2012. Morb. Mortal. Wkly. Rep. 62, 405–408 PMC4604938 23698606

[4] Mackenzie C. D., Homeida M. M., Hopkins A. D., and Lawrence J. C. (2012) Elimination of onchocerciasis from Africa: possible? Trends Parasitol. 28, 16–22 22079526 10.1016/j.pt.2011.10.003

[5] Zimmerman P. A., Guderian R. H., Aruajo E., Elson L., Phadke P., Kubofcik J., and Nutman T. B. (1994) Polymerase chain reaction-based diagnosis of Onchocerca volvulus infection: improved detection of patients with onchocerciasis. J. Infect. Dis. 169, 686–689 8158053 10.1093/infdis/169.3.686

[6] Lobos E., Weiss N., Karam M., Taylor H. R., Ottesen E. A., and Nutman T. B. (1991) An immunogenic Onchocerca volvulus antigen: a specific and early marker of infection. Science 251, 1603–1605 2011741 10.1126/science.2011741

[7] Bradley J. E., Trenholme K. R., Gillespie A. J., Guderian R., Titanji V., Hong Y., and McReynolds L. (1993) A sensitive serodiagnostic test for onchocerciasis using a cocktail of recombinant antigens. Am. J. Trop. Med. Hyg. 48, 198–204 8447523 10.4269/ajtmh.1993.48.198

[8] Ogunrinade A. F., Chandrashekar R., Weil G. J., and Kale O. O. (1992) Use of a recombinant antigen (OC 3.6 cDNA) for the serological diagnosis of onchocerciasis in exposed Nigerian children. J. Trop. Pediatr. 38, 103–105 1507301 10.1093/tropej/38.3.103

[9] Lucius R., Kern A., Seeber F., Pogonka T., Willenbucher J., Taylor H. R., Pinder M., Ghalib H. W., Schulz-Key H., and Soboslay P. (1992) Specific and sensitive IgG4 immunodiagnosis of onchocerciasis with a recombinant 33 kD Onchocerca volvulus protein (Ov33). Trop. Med. Parasitol. 43, 139–145 1281926

[10] Lipner E. M., Dembele N., Souleymane S., Alley W. S., Prevots D. R., Toe L., Boatin B., Weil G. J., and Nutman T. B. (2006) Field applicability of a rapid-format anti-Ov-16 antibody test for the assessment of onchocerciasis control measures in regions of endemicity. J. Infect. Dis. 194, 216–221 16779728 10.1086/505081

[11] Weil G. J., Steel C., Liftis F., Li B. W., Mearns G., Lobos E., and Nutman T. B. (2000) A rapid-format antibody card test for diagnosis of onchocerciasis. J. Infect. Dis. 182, 1796–1799 11069258 10.1086/317629

[12] Ambroise-Thomas P. (1974) Immunological diagnosis of human filariases: present possibilities, difficulties and limitations. Acta Trop. 31, 108–128 4152907

[13] Marcoullis G., and Grasbeck R. (1976) Preliminary identification and characterization of antigen extracts from Onchocerca volvulus. Tropenmed. Parasitol. 27, 314–322 824771

[14] Klenk A., Geyer E., and Zahner H. (1984) Serodiagnosis of human onchocerciasis: evaluation of sensitivity and specificity of a purified Litomosoides carinii adult worm antigen. Tropenmed. Parasitol. 35, 81–84 6205486

[15] Lavebratt C., Dalhammar G., Adamafio N. A., Nykanen-Dejerud U., Mingarini K., Ingemarsson K., Opoku N., and Akuffo H. O. (1994) A simple dot blot assay adaptable for field use in the diagnosis of onchocerciasis: preparation of an adult worm antigen fraction which enhances sensitivity and specificity. Trans. R. Soc. Trop. Med. Hyg. 88, 303–306 7974670 10.1016/0035-9203(94)90090-6

[16] Tada I., Korenaga M., Shiwaku K., Ogunba E. O., Ufomadu G. O., and Nwoke B. E. (1987) Specific serodiagnosis with adult Onchocerca volvulus antigen in an enzyme-linked immunosorbent assay. Am. J. Trop. Med. Hyg. 36, 383–386 3469920 10.4269/ajtmh.1987.36.383

[17] Lobos E., and Weiss N. (1986) Identification of non-cross-reacting antigens of Onchocerca volvulus with lymphatic filariasis serum pools. Parasitology 93 (Pt 2), 389–399 3785977 10.1017/s0031182000051556

[18] Unnasch T. R., Gallin M. Y., Soboslay P. T., Erttmann K. D., and Greene B. M. (1988) Isolation and characterization of expression cDNA clones encoding antigens of Onchocerca volvulus infective larvae. J. Clin. Invest. 82, 262–269 2455736 10.1172/JCI113581 PMC303503

[19] Lustigman S., Brotman B., Johnson E. H., Smith A. B., Huima T., and Prince A. M. (1992) Identification and characterization of an Onchocerca volvulus cDNA clone encoding a microfilarial surface-associated antigen. Mol. Biochem. Parasitol. 50, 79–93 1542318 10.1016/0166-6851(92)90246-g

[20] Chandrashekar R., Masood K., Alvarez R. M., Ogunrinade A. F., Lujan R., Richards F. O. Jr., and Weil G. J. (1991) Molecular cloning and characterization of recombinant parasite antigens for immunodiagnosis of onchocerciasis. J. Clin. Invest. 88, 1460–1466 1840605 10.1172/JCI115455 PMC295649

[21] Bradley J. E., Tuan R. S., Shepley K. J., Tree T. I., Maizels R. M., Helm R., Gregory W. F., and Unnasch T. R. (1993) Onchocerca volvulus: characterization of an immunodominant hypodermal antigen present in adult and larval parasites. Exp. Parasitol. 77, 414–424 8253155 10.1006/expr.1993.1101

[22] Nde P. N., Pogonka T., Bradley J. E., Titanji V. P., and Lucius R. (2002) Sensitive and specific serodiagnosis of onchocerciasis with recombinant hybrid proteins. Am. J. Trop. Med. Hyg. 66, 566–571 12201591 10.4269/ajtmh.2002.66.566

[23] Golden A., Steel C., Yokobe L., Jackson E., Barney R., Kubofcik J., Peck R., Unnasch T. R., Nutman T. B., de los Santos T., and Domingo G. J. (2013) Extended result reading window in lateral flow tests detecting exposure to Onchocerca volvulus: a new technology to improve epidemiological surveillance tools. PloS one 8, e69231 23935960 10.1371/journal.pone.0069231 PMC3720650

[24] Gallin M. Y., Tan M., Kron M. A., Rechnitzer D., Greene B. M., Newland H. S., White A. T., Taylor H. R., and Unnasch T. R. (1989) Onchocerca volvulus recombinant antigen: physical characterization and clinical correlates with serum reactivity. J. Infect. Dis. 160, 521–529 2760503 10.1093/infdis/160.3.521

[25] Ajonina-Ekoti I., Ndjonka D., Tanyi M. K., Wilbertz M., Younis A. E., Boursou D., Kurosinski M. A., Eberle R., Luersen K., Perbandt M., Breloer M., Brattig N. W., and Liebau E. (2012) Functional characterization and immune recognition of the extracellular superoxide dismutase from the human pathogenic parasite Onchocerca volvulus (OvEC-SOD). Acta Trop. 124, 15–26 22677600 10.1016/j.actatropica.2012.05.013

[26] Mpagi J. L., Buttner D. W., Tischendorf F. W., Erttmann K. D., and Brattig N. W. (2000) Humoral responses to a secretory Onchocerca volvulus protein: differences in the pattern of antibody isotypes to recombinant Ov20/OvS1 in generalized and hyperreactive onchocerciasis. Parasite Immunol. 22, 455–460 10972852 10.1046/j.1365-3024.2000.00325.x

[27] Wanni N. O., Strote G., Rubaale T., and Brattig N. W. (1997) Demonstration of immunoglobulin G antibodies against Onchocerca volvulus excretory-secretory antigens in different forms and stages of onchocerciasis. Trans. R. Soc. Trop. Med. Hyg. 91, 226–230 9196778 10.1016/s0035-9203(97)90234-0

[28] Camacho C., Coulouris G., Avagyan V., Ma N., Papadopoulos J., Bealer K., and Madden T. L. (2009) BLAST+: architecture and applications. BMC Bioinformatics 10, 421 20003500 10.1186/1471-2105-10-421 PMC2803857

[29] Tang Y. T., Gao X., Rosa B. A., Abubucker S., Hallsworth-Pepin K., Martin J., Tyagi R., Heizer E., Zhang X., Bhonagiri-Palsikar V., Minx P., Warren W. C., Wang Q., Zhan B., Hotez P. J., Sternberg P. W., Dougall A., Gaze S. T., Mulvenna J., Sotillo J., Ranganathan S., Rabelo E. M., Wilson R. K., Felgner P. L., Bethony J., Hawdon J. M., Gasser R. B., Loukas A., and Mitreva M. (2014) Genome of the human hookworm Necator americanus. Nature Genetics 46, 261–269. 24441737 10.1038/ng.2875 PMC3978129

[30] Sievers F., Wilm A., Dineen D., Gibson T. J., Karplus K., Li W., Lopez R., McWilliam H., Remmert M., Soding J., Thompson J. D., and Higgins D. G. (2011) Fast, scalable generation of high-quality protein multiple sequence alignments using Clustal Omega. Mol. Syst. Biol. 7, 539 21988835 10.1038/msb.2011.75 PMC3261699

[31] Kall L., Krogh A., and Sonnhammer E. L. (2004) A combined transmembrane topology and signal peptide prediction method. J. Mol. Biol. 338, 1027–1036 15111065 10.1016/j.jmb.2004.03.016

[32] Kall L., Krogh A., and Sonnhammer E. L. (2007) Advantages of combined transmembrane topology and signal peptide prediction–the Phobius web server. Nucleic Acids Res. 35, W429–432 17483518 10.1093/nar/gkm256 PMC1933244

[33] Bendtsen J. D., Jensen L. J., Blom N., Von Heijne G., and Brunak S. (2004) Feature-based prediction of non-classical and leaderless protein secretion. Protein Eng. Des. Sel. 17, 349–356 15115854 10.1093/protein/gzh037

[34] Rawlings N. D., Waller M., Barrett A. J., and Bateman A. (2014) MEROPS: the database of proteolytic enzymes, their substrates and inhibitors. Nucleic Acids Res. 42, 23 10.1093/nar/gkt953 PMC3964991 24157837

[35] Wylie T., Martin J., Abubucker S., Yin Y., Messina D., Wang Z., McCarter J. P., and Mitreva M. (2008) NemaPath: online exploration of KEGG-based metabolic pathways for nematodes. BMC Gen. 9, 525 10.1186/1471-2164-9-525 PMC2588608 18983679

[36] Kanehisa M., Goto S., Sato Y., Furumichi M., and Tanabe M. (2012) KEGG for integration and interpretation of large-scale molecular data sets. Nucleic Acids Res. 40, D109–114 22080510 10.1093/nar/gkr988 PMC3245020

[37] Ashburner M., Ball C. A., Blake J. A., Botstein D., Butler H., Cherry J. M., Davis A. P., Dolinski K., Dwight S. S., Eppig J. T., Harris M. A., Hill D. P., Issel-Tarver L., Kasarskis A., Lewis S., Matese J. C., Richardson J. E., Ringwald M., Rubin G. M., and Sherlock G. (2000) Gene ontology: tool for the unification of biology. The Gene Ontology Consortium. Nat. Genet. 25, 25–29 10802651 10.1038/75556 PMC3037419

[38] Hunter S., Jones P., Mitchell A., Apweiler R., Attwood T. K., Bateman A., Bernard T., Binns D., Bork P., Burge S., de Castro E., Coggill P., Corbett M., Das U., Daugherty L., Duquenne L., Finn R. D., Fraser M., Gough J., Haft D., Hulo N., Kahn D., Kelly E., Letunic I., Lonsdale D., Lopez R., Madera M., Maslen J., McAnulla C., McDowall J., McMenamin C., Mi H., Mutowo-Muellenet P., Mulder N., Natale D., Orengo C., Pesseat S., Punta M., Quinn A. F., Rivoire C., Sangrador-Vegas A., Selengut J. D., Sigrist C. J., Scheremetjew M., Tate J., Thimmajanarthanan M., Thomas P. D., Wu C. H., Yeats C., and Yong S. Y. (2012) InterPro in 2011: new developments in the family and domain prediction database. Nucleic Acids Res. 40, D306–312 22096229 10.1093/nar/gkr948 PMC3245097

[39] Quevillon E., Silventoinen V., Pillai S., Harte N., Mulder N., Apweiler R., and Lopez R. (2005) InterProScan: protein domains identifier. Nucleic Acids Res. 33, W116–120 15980438 10.1093/nar/gki442 PMC1160203

[40] Arndts K., Specht S., Debrah A. Y., Tamarozzi F., Klarmann Schulz U., Mand S., Batsa L., Kwarteng A., Taylor M., Adjei O., Martin C., Layland L. E., and Hoerauf A. (2014) Immunoepidemiological profiling of onchocerciasis patients reveals associations with microfilaria loads and ivermectin intake on both individual and community levels. PLoS Neglected Trop. Dis. 8, e2679 10.1371/journal.pntd.0002679 PMC3930501 24587458

[41] McNulty S. N., Fischer P. U., Townsend R. R., Curtis K. C., Weil G. J., and Mitreva M. (2014) Systems biology studies of adult paragonimus lung flukes facilitate the identification of immunodominant parasite antigens. PLoS Negl Trop. Dis. 8, e3242 25329661 10.1371/journal.pntd.0003242 PMC4199545

[42] Kim D., Pertea G., Trapnell C., Pimentel H., Kelley R., and Salzberg S. L. (2013) TopHat2: accurate alignment of transcriptomes in the presence of insertions, deletions and gene fusions. Genome Biol. 14, R36 23618408 10.1186/gb-2013-14-4-r36 PMC4053844

[43] Lee J. E., Kellie J. F., Tran J. C., Tipton J. D., Catherman A. D., Thomas H. M., Ahlf D. R., Durbin K. R., Vellaichamy A., Ntai I., Marshall A. G., and Kelleher N. L. (2009) A robust two-dimensional separation for top-down tandem mass spectrometry of the low-mass proteome. J. Am. Soc. Mass Spectrom. 20, 2183–2191 19747844 10.1016/j.jasms.2009.08.001 PMC2830800

[44] Orton D. J., Arsenault D. J., Thomas N. A., and Doucette A. A. (2013) GELFrEE fractionation combined with mass spectrometry for proteome analysis of secreted toxins from Enteropathogenic Escherichia coli (EPEC). Mol. Cell. Probes 27, 200–207 23831145 10.1016/j.mcp.2013.06.004

[45] Rost H., Malmstrom L., and Aebersold R. (2012) A computational tool to detect and avoid redundancy in selected reaction monitoring. Mol. Cell. Proteomics 11, 540–549 22535207 10.1074/mcp.M111.013045 PMC3412981

[46] Wisniewski J. R., Zougman A., Nagaraj N., and Mann M. (2009) Universal sample preparation method for proteome analysis. Nature Methods 6, 359–362 19377485 10.1038/nmeth.1322

[47] Zhang J., Xin L., Shan B., Chen W., Xie M., Yuen D., Zhang W., Zhang Z., Lajoie G. A., and Ma B. (2012) PEAKS DB: de novo sequencing assisted database search for sensitive and accurate peptide identification. Mol. Cell. Proteomics : MCP 11, M111 010587 10.1074/mcp.M111.010587 PMC3322562 22186715

[48] Anders S., and Huber W. (2010) Differential expression analysis for sequence count data. Genome Biology 11, R106 20979621 10.1186/gb-2010-11-10-r106 PMC3218662

[49] Benjamini Y., and Hochberg Y. (1995) Controlling the False Discovery Rate: A Practical and Powerful Approach to Multiple Testing. J. Roy. Statistical Soc. 57, 289–300

[50] Bennuru S., Semnani R., Meng Z., Ribeiro J. M., Veenstra T. D., and Nutman T. B. (2009) Brugia malayi excreted/secreted proteins at the host/parasite interface: stage- and gender-specific proteomic profiling. PLoS Negl Trop. Dis. 3, e410 19352421 10.1371/journal.pntd.0000410 PMC2659452

[51] McNulty S. N., Abubucker S., Simon G. M., Mitreva M., McNulty N. P., Fischer K., Curtis K. C., Brattig N. W., Weil G. J., and Fischer P. U. (2012) Transcriptomic and proteomic analyses of a Wolbachia-free filarial parasite provide evidence of trans-kingdom horizontal gene transfer. PLoS ONE 7, e45777 23049857 10.1371/journal.pone.0045777 PMC3458923

[52] Bennuru S., Meng Z., Ribeiro J. M., Semnani R. T., Ghedin E., Chan K., Lucas D. A., Veenstra T. D., and Nutman T. B. (2011) Stage-specific proteomic expression patterns of the human filarial parasite Brugia malayi and its endosymbiont Wolbachia. Proc. Natl. Acad. Sci. U.S.A. 108, 9649–9654 21606368 10.1073/pnas.1011481108 PMC3111283

[53] Rosa B. A., Townsend R., Jasmer D. P., and Mitreva M. (2015) Functional and phylogenetic characterization of proteins detected in various nematode intestinal compartments. Mol. Cell. Proteomics 14, 812–827 25609831 10.1074/mcp.M114.046227 PMC4390262

[54] Foss E. J., Radulovic D., Shaffer S. A., Ruderfer D. M., Bedalov A., Goodlett D. R., and Kruglyak L. (2007) Genetic basis of proteome variation in yeast. Nat. Genet. 39, 1369–1375 17952072 10.1038/ng.2007.22

[55] Lal R. B., and Ottesen E. A. (1988) Enhanced diagnostic specificity in human filariasis by IgG4 antibody assessment. J. Infect. Dis. 158, 1034–1037 2460565 10.1093/infdis/158.5.1034

[56] Chandrashekar R., Curtis K. C., Ramzy R. M., Liftis F., Li B. W., and Weil G. J. (1994) Molecular cloning of Brugia malayi antigens for diagnosis of lymphatic filariasis. Mol. Biochem. Parasitol. 64, 261–271 7935604 10.1016/0166-6851(94)00035-2

[57] Weil G. J., Curtis K. C., Fischer P. U., Won K. Y., Lammie P. J., Joseph H., Melrose W. D., and Brattig N. W. (2011) A multicenter evaluation of a new antibody test kit for lymphatic filariasis employing recombinant Brugia malayi antigen Bm-14. Acta Trop. 120, S19–22 20430004 10.1016/j.actatropica.2010.04.010 PMC2935504

[58] Lustigman S., Brotman B., Huima T., and Prince A. M. (1991) Characterization of an Onchocerca volvulus cDNA clone encoding a genus specific antigen present in infective larvae and adult worms. Mol. Biochem. Parasitol. 45, 65–75 2052041 10.1016/0166-6851(91)90028-5

[59] Chandrashekar R., Ogunrinade A. F., and Weil G. J. (1996) Use of recombinant Onchocerca volvulus antigens for diagnosis and surveillance of human onchocerciasis. Trop. Med. Int. Health 1, 575–580 8911441 10.1111/j.1365-3156.1996.tb00082.x

[60] Ogunrinade A. F., Chandrashekar R., Eberhard M. L., and Weil G. J. (1993) Preliminary evaluation of recombinant Onchocerca volvulus antigens for serodiagnosis of onchocerciasis. Journal of clinical microbiology 31, 1741–1745 8349749 10.1128/jcm.31.7.1741-1745.1993 PMC265624

[61] Eberhard M. L., Dickerson J. W., Tsang V. C., Walker E. M., Ottesen E. A., Chandrashekar R., Weil G. J., Trpis M., Strobert E., Constantinidis I., and et al (1995) Onchocerca volvulus: parasitologic and serologic responses in experimentally infected chimpanzees and mangabey monkeys. Exp. Parasitol. 80, 454–462 7729480 10.1006/expr.1995.1057

[62] Chandrashekar R., Ogunrinade A. F., Alvarez R. M., Kale O. O., and Weil G. J. (1990) Circulating immune complex-associated parasite antigens in human onchocerciasis. J. Infect. Dis. 162, 1159–1164 2230240 10.1093/infdis/162.5.1159

[63] Henkle K. J., Liebau E., Muller S., Bergmann B., and Walter R. D. (1991) Characterization and molecular cloning of a Cu/Zn superoxide dismutase from the human parasite Onchocerca volvulus. Infect. Immun. 59, 2063–2069 2037366 10.1128/iai.59.6.2063-2069.1991 PMC257966

[64] Bradley J. E., Helm R., Lahaise M., and Maizels R. M. (1991) cDNA clones of Onchocerca volvulus low molecular weight antigens provide immunologically specific diagnostic probes. Mol. Biochem. Parasitol. 46, 219–227 1922197 10.1016/0166-6851(91)90046-9

[65] Irvine M., Huima T., Prince A. M., and Lustigman S. (1994) Identification and characterization of an Onchocerca volvulus cDNA clone encoding a highly immunogenic calponin-like protein. Mol. Biochem. Parasitol. 65, 135–146 7935620 10.1016/0166-6851(94)90122-8

[66] McCarthy J. S., Wieseman M., Tropea J., Kaslow D., Abraham D., Lustigman S., Tuan R., Guderian R. H., and Nutman T. B. (2002) Onchocerca volvulus glycolytic enzyme fructose-1,6-bisphosphate aldolase as a target for a protective immune response in humans. Infect. Immun. 70, 851–858 11796620 10.1128/IAI.70.2.851-858.2002 PMC127653

[67] Jolodar A., Fischer P., Bergmann S., Buttner D. W., Hammerschmidt S., and Brattig N. W. (2003) Molecular cloning of an alpha-enolase from the human filarial parasite Onchocerca volvulus that binds human plasminogen. Biochim. Biophys. Acta 1627, 111–120 12818429 10.1016/s0167-4781(03)00083-6

[68] Taylor M. J., Abdel-Wahab N., Wu Y., Jenkins R. E., and Bianco A. E. (1995) Onchocerca volvulus larval antigen, OvB20, induces partial protection in a rodent model of onchocerciasis. Infect. Immun. 63, 4417–4422 7591079 10.1128/iai.63.11.4417-4422.1995 PMC173628

[69] Graham S. P., Lustigman S., Trees A. J., and Bianco A. E. (2000) Onchocerca volvulus: comparative analysis of antibody responses to recombinant antigens in two animal models of onchocerciasis. Exp. Parasitol. 94, 158–162 10831380 10.1006/expr.2000.4487

[70] Taylor M. J., Jenkins R. E., and Bianco A. E. (1996) Protective immunity induced by vaccination with Onchocerca volvulus tropomyosin in rodents. Parasite Immunol. 18, 219–225 9229374 10.1046/j.1365-3024.1996.d01-93.x

[71] Jenkins R. E., Taylor M. J., Gilvary N. J., and Bianco A. E. (1998) Tropomyosin implicated in host protective responses to microfilariae in onchocerciasis. Proc. Natl. Acad. Sci. U.S.A. 95, 7550–7555 9636187 10.1073/pnas.95.13.7550 PMC22680

[72] Liebau E., Wildenburg G., Walter R. D., and Henkle-Duhrsen K. (1994) A novel type of glutathione S-transferase in Onchocerca volvulus. Infect. Immun. 62, 4762–4767 7927752 10.1128/iai.62.11.4762-4767.1994 PMC303184

[73] Graham S. P., Wu Y., Henkle-Duehrsen K., Lustigman S., Unnasch T. R., Braun G., Williams S. A., McCarthy J., Trees A. J., and Bianco A. E. (1999) Patterns of Onchocerca volvulus recombinant antigen recognition in a bovine model of onchocerciasis. Parasitology 119, 603–612 10633922 10.1017/s0031182099005065

[74] Triteeraprapab S., Richie T. L., Tuan R. S., Shepley K. J., Dinman J. D., Neubert T. A., and Scott A. L. (1995) Molecular cloning of a gene expressed during early embryonic development in Onchocerca volvulus. Mol. Biochem. Parasitol. 69, 161–171 7770081 10.1016/0166-6851(94)00187-r

[75] Joseph G. T., Huima T., and Lustigman S. (1998) Characterization of an Onchocerca volvulus L3-specific larval antigen, Ov-ALT-1. Mol. Biochem. Parasitol. 96, 177–183 9851616 10.1016/s0166-6851(98)00094-2

[76] Tawe W., Pearlman E., Unnasch T. R., and Lustigman S. (2000) Angiogenic activity of Onchocerca volvulus recombinant proteins similar to vespid venom antigen 5. Mol. Biochem. Parasitol. 109, 91–99 10960168 10.1016/s0166-6851(00)00231-0

[77] MacDonald A. J., Tawe W., Leon O., Cao L., Liu J., Oksov Y., Abraham D., and Lustigman S. (2004) Ov-ASP-1, the Onchocerca volvulus homologue of the activation associated secreted protein family is immunostimulatory and can induce protective anti-larval immunity. Parasite Immunol. 26, 53–62 15198646 10.1111/j.0141-9838.2004.00685.x

[78] Wu Y., Adam R., Williams S. A., and Bianco A. E. (1996) Chitinase genes expressed by infective larvae of the filarial nematodes, Acanthocheilonema viteae and Onchocerca volvulus. Mol. Biochem. Parasitol. 75, 207–219 8992319 10.1016/0166-6851(95)02529-4

[79] Wu Y., Egerton G., McCarthy J. S., Nutman T. B., and Bianco A. E. (2003) Human immune responses to infective stage larval-specific chitinase of filarial parasite, Onchocerca volvulus, Ov-CHI-1. Filaria J 2, 6 12685941 10.1186/1475-2883-2-6 PMC153484

[80] Abraham D., Leon O., Leon S., and Lustigman S. (2001) Development of a recombinant antigen vaccine against infection with the filarial worm Onchocerca volvulus. Infect. Immun. 69, 262–270 11119514 10.1128/IAI.69.1.262-270.2001 PMC97880

[81] Park J., Dickerson T. J., and Janda K. D. (2008) Major sperm protein as a diagnostic antigen for onchocerciasis. Bioorg. Med. Chem. 16, 7206–7209 18632276 10.1016/j.bmc.2008.06.038

[82] Scott A. L., Dinman J., Sussman D. J., Yenbutr P., and Ward S. (1989) Major sperm protein genes from Onchocerca volvulus. Mol. Biochem. Parasitol. 36, 119–126 2770787 10.1016/0166-6851(89)90184-9

