# Supplementary material for: An Integrated Multiomics Approach to Identify Candidate Antigens for Serodiagnosis of Human Onchocerciasis
Source: Mol Cell Proteomics. 2015 Oct 15;14(12):3224–33. doi: 10.1074/mcp.M115.051953 (PMC4762623; doi:10.1074/mcp.M115.051953)
Supplement: Supplemental Data [file supp_14_12_3224__index.html]

An Integrated Multiomics Approach to Identify Candidate Antigens for Serodiagnosis of Human Onchocerciasis — Identification of Serodiagnostic Antigens for Onchocerciasis — Supplemental Data 

# An Integrated Multiomics Approach to Identify Candidate Antigens for Serodiagnosis of Human Onchocerciasis

## Supplemental Data

**Files in this Data Supplement:**

- Supplemental Table S2
- Supplemental Table S3
- Dataset S1
- Dataset S2
- Supplemental Figure S1
- Supplemental Figure S3
- Supplemental Figure S4
- Supplemental Figure S5
- Supplemental Figure 2
- Supplemental Table S1
