# Supplementary material for: An Integrated Multiomics Approach to Identify Candidate Antigens for Serodiagnosis of Human Onchocerciasis
Source: Mol Cell Proteomics. 2015 Oct 15;14(12):3224–33. doi: 10.1074/mcp.M115.051953 (PMC4762623; doi:10.1074/mcp.M115.051953)
Supplement: Supplemental Data [file supp_M115.051953_Table_S2.pdf]

Table S2. Summary of Illumina HiSeq 2000 cDNA libraries sequenced for the present study*.

SRA Experiment Accession	Stage	Raw Read Pairs
SRX978164	Adult female	29,870,749
SRX978163	Adult female	32,228,192
SRX978161	Adult female	18,853,485
SRX978160	Adult female	27,441,004
SRX978159	Adult female	24,730,365
SRX978158	Adult female	22,830,107
SRX978157	Adult female	27,131,675
SRX978156	Adult female	28,781,766
SRX978162	Adult male	38,964,547

*All libraries accession numbers are related to GenBank BioProject PRJNA219638.
