# Supplementary material for: An Integrated Multiomics Approach to Identify Candidate Antigens for Serodiagnosis of Human Onchocerciasis
Source: Mol Cell Proteomics. 2015 Oct 15;14(12):3224–33. doi: 10.1074/mcp.M115.051953 (PMC4762623; doi:10.1074/mcp.M115.051953)
Supplement: Supplemental Data [file supp_M115.051953_Table_S3.pdf]

Table S5. Characterization of 60 immunoreactive, *Onchocerca volvulus*-specific proteins

Gene	Top KEGG orthologous group annotation	RNAseq expression (FPKM)				Protein length (aa)	Expression-based Prioritization	Fractionated whole-worm lysate spectral count	Top BLAST hit		
		Fe-male	Male	L3	MF				Species	%ID	%Length
OVOC9748	GALNT; polypeptide N-acetylgalactosaminyltransferase	99	412	47	2	2,971	Y	7,487	*L.loa*	72	57.5
OVOC10067	K08473; nematode chemoreceptor	25	40	36	5	3,415	Y	2,079	*S.stercoralis*	43.6	98.4
OVOC9475	SUMO, SMT3; small ubiquitin-related modifier	701	810	105	15	1,322	Y	2,242	*A.lumbricoides*	80.7	45.6
OVOC9752	acpS; holo-[acyl-carrier protein] synthase	1,631	596	4,510	1,296	467	Y	1,635	*W.bancrofti*	94	42.6
OVOC8985	GGPS; geranylgeranyl diphosphate synthase, type II	546	291	263	490	710	Y	1,296	*A.lumbricoides*	62.2	97.5
OVOC11951	TRIM9_67; tripartite motif-containing protein 9/67	169	98	1	1	230	Y	1,082	*B.malayi*	69.9	97
OVOC9325	KIDINS220, ARMS; ankyrin repeat-rich membrane spanning protein	2,492	331	2,977	989	365	Y	825	*A.lumbricoides*	49.8	85.2
OVOC7381	EPRS; bifunctional glutamyl/prolyl-tRNA synthetase	475	435	231	179	683	Y	642	*L.loa*	68.1	92.8
OVOC10103	ECE; endothelin-converting enzyme	52	15	79	17	828	Y	462	*S.stercoralis*	49.8	97.8
OVOC9592	SNRP70; U1 small nuclear ribonucleoprotein 70kDa	159	175	609	904	279	Y	545	*A.lumbricoides*	59.1	98.2
OVOC4612	PRCC; proline-rich protein PRCC	1,503	792	83	3	257	Y	411	*N.americanus*	45.1	82.9
OVOC12400	PRPF31; U4/U6 small nuclear ribonucleoprotein PRP31	507	280	91	7	147	Y	389	*W.bancrofti*	69.4	100
OVOC7453	groEL, HSPD1; chaperonin GroEL	1,415	1,668	13,640	539	162	Y	429	*B.malayi*	66.2	98.8
OVOC12449	POU3F, OTF; POU domain transcription factor, class 3	1,227	1	1	0	249	Y	432	*W.bancrofti*	51.4	96
OVOC3203	CAP1_2, SRV2; adenylyl cyclase-associated protein	25	41	66	49	1,085	Y	359	*A.lumbricoides*	42.2	99.9
OVOC5823	DCTN5; dynactin 5	124	66	414	189	593	Y	291	*W.bancrofti*	99	49.2
OVOC9988	ROR2, NTRKR2; receptor tyrosine kinase-like orphan receptor 2	4,008	3,597	4,819	1,914	164	Y	370	*B.malayi*	57.7	99.4
OVOC2486	NDUFA1; NADH dehydrogenase (ubiquinone) 1 alpha subcomplex subu	365	514	708	81	518	Y	240	*B.malayi*	41.8	97.5
OVOC1213	EDD1, UBR5; E3 ubiquitin-protein ligase EDD1	72	17	395	9	677	Y	218	*A.lumbricoides*	31.6	91.1
OVOC11487	GAPDH, gapA; glyceraldehyde 3-phosphate dehydrogenase	301	120	163	113	211	Y	190	*L.loa*	69.9	98.1
OVOC6327	MYO5; myosin V	146	180	604	291	147	Y	152	*B.malayi*	67.3	100
OVOC11847	NOVA; RNA-binding protein Nova	909	790	440	989	456	Y	149	*L.loa*	50.2	100
OVOC1897	RP-L5e, RPL5; large subunit ribosomal protein L5e	40	139	97	2	963	Y	91	*B.malayi*	52.9	99.1
OVOC5718	RBM7; RNA-binding protein 7	6,292	5	0	0	104	Y	105	*B.malayi*	90.8	62.5
OVOC9990	ROR2, NTRKR2; receptor tyrosine kinase-like orphan receptor 2	625	335	773	4	192	Y	90	*H.sapiens*	29.5	83.3
OVOC5419	HSD17B10; 3-hydroxyacyl-CoA dehydrogenase / 3-hydroxy-2-methylbut	152	43	75	138	580	Y	70	*L.loa*	92.2	44.5
OVOC12448	SF3A1, SAP114; splicing factor 3A subunit 1	454	0	0	0	155	Y	47	*L.loa*	55.8	99.4
OVOC10995	ALDH5A1; succinate-semialdehyde dehydrogenase	5,798	17	143	86	131	Y	54	*W.bancrofti*	47.8	99.2
OVOC7430	SOS; son of sevenless	90	34	69	2	185	Y	42	*B.malayi*	61.2	78.4
OVOC10638	APPBP1; amyloid beta precursor protein binding protein 1	634	984	83	1	359	Y	16	*B.malayi*	56.3	78.3
OVOC11218	glgB; 1,4-alpha-glucan branching enzyme	142	112	390	197	215	Y	13	*B.malayi*	66.1	100
OVOC10982	ARFGAP2_3; ADP-ribosylation factor GTPase-activating protein 2/3	31	3	709	154	190	Y	5	*W.bancrofti*	56.6	87.4
OVOC9384	MRD1, RBM19; multiple RNA-binding domain-containing protein 1	6,925	14,337	1,850	655	746	Y	2	*L.loa*	51.2	100
OVOC8391	TAF1; transcription initiation factor TFIID subunit 1	10	61	30	10	6,568	-	1110	*B.malayi*	65.6	100
OVOC10654	MALT1; mucosa-associated lymphoid tissue lymphoma translocation pro	286	68	128	10	1,729	-	1001	*B.malayi*	46.1	99.9
OVOC451	ELF1_2_4; E74-like factor 1/2/4	1,522	721	489	683	209	-	637	*L.loa*	49.4	73.7
OVOC8665	GST, gst; glutathione S-transferase	496	2	4	1	265	-	625	*B.malayi*	55.3	100
OVOC2358	PTCH1; patched 1	12	72	1	0	539	-	309	*L.loa*	50.2	89.4
OVOC1838	EDEM2; ER degradation enhancer, mannosidase alpha-like 2	28	1	237	2	526	-	315	*L.loa*	63.9	98.7
OVOC7082	CALM; calmodulin	418	131	1,419	244	201	-	279	*B.malayi*	62.2	100
OVOC10529	-	145	3,083	0	1	106	-	258	*L.loa*	35.6	80.2
OVOC9989	ROR2, NTRKR2; receptor tyrosine kinase-like orphan receptor 2	48,769	0	8	5	139	-	220	*B.malayi*	45.1	100
OVOC11516	SOD1; superoxide dismutase, Cu-Zn family	996	347	3,494	95	201	-	185	*B.malayi*	63.9	77.1
OVOC10221	NOS1AP, CAPON; carboxyl-terminal PDZ ligand of neuronal nitric oxide	63	1	1	2	267	-	173	*W.bancrofti*	37.8	86.5
OVOC7777	PPP1C; serine/threonine-protein phosphatase PP1 catalytic subunit	69	25	2,329	23	129	-	135	*W.bancrofti*	59.7	100
OVOC2673	RABL5; Rab-like protein 5	28	794	22	18	221	-	85	*A.lumbricoides*	56.2	97.7
OVOC387	RRP12; ribosomal RNA-processing protein 12	3,762	2	300	1	119	-	97	*L.loa*	50	100
OVOC12688	PMM; phosphomannomutase	753	95	840	1	157	-	79	*B.malayi*	66	95.5
OVOC11213	SNRPD1, SMD1; small nuclear ribonucleoprotein D1	34	53	47	183	256	-	53	*W.bancrofti*	64.9	100
OVOC9514	E2.3.1.97, NMT; glycylpeptide N-tetradecanoyltransferase	29	10	44	16	140	-	43	*B.malayi*	60	100
OVOC7606	DDX5, DBP2; ATP-dependent RNA helicase DDX5/DBP2	549	0	1	47	210	-	32	*B.malayi*	43.3	92.9
OVOC3924	ROR2, NTRKR2; receptor tyrosine kinase-like orphan receptor 2	34	8	5	9	334	-	25	*B.malayi*	69.3	100
OVOC8491	MYL12; myosin regulatory light chain 12	25	24	307	1,083	253	-	35	*W.bancrofti*	63.5	98.8
OVOC9058	TM9SF2_4; transmembrane 9 superfamily member 2/4	10	6	2	546	182	-	21	*W.bancrofti*	65.5	97.3
OVOC422	AXIN1; axin 1	90	3	1	2	327	-	19	*L.loa*	66.4	99.7
OVOC12544	PRCP; lysosomal Pro-X carboxypeptidase	288	1	0	0	96	-	13	*L.loa*	55	100
OVOC12586	TUBA; tubulin alpha	173	0	0	0	72	-	12	*S.stercoralis*	60	62.5
OVOC6329	E1.6.2.2; cytochrome-b5 reductase	23	15	19	8	588	-	9	*B.malayi*	60.4	78.9
OVOC12404	atuD; citronellyl-CoA dehydrogenase	144	4	0	0	98	-	8	*B.malayi*	60	96.9
OVOC2080	SERPINB; serpin B	4	2	273	17	362	-	7	*B.malayi*	56.9	99.4
