# Supplementary material for: An Integrated Multiomics Approach to Identify Candidate Antigens for Serodiagnosis of Human Onchocerciasis
Source: Mol Cell Proteomics. 2015 Oct 15;14(12):3224–33. doi: 10.1074/mcp.M115.051953 (PMC4762623; doi:10.1074/mcp.M115.051953)
Supplement: Supplemental Data [file supp_M115.051953_Figure_S1.docx]

**Figure S1: Conservation of inferred *O. volvulus* proteins with orthologs from relevant species.**

A heatmap displaying the number of *O. volvulus* genes which are identified as *O. volvulus*-specific according to BLAST cutoffs for the percentage of the query covered, and the percent identity shared with the top scoring subject. For prioritization, a cutoff of 70%/70% was chosen, identifying 4,753 *O. volvulus*-specific proteins.
