# Supplementary material for: An Integrated Multiomics Approach to Identify Candidate Antigens for Serodiagnosis of Human Onchocerciasis
Source: Mol Cell Proteomics. 2015 Oct 15;14(12):3224–33. doi: 10.1074/mcp.M115.051953 (PMC4762623; doi:10.1074/mcp.M115.051953)
Supplement: Supplemental Data [file supp_M115.051953_Figure_S3.docx]

**
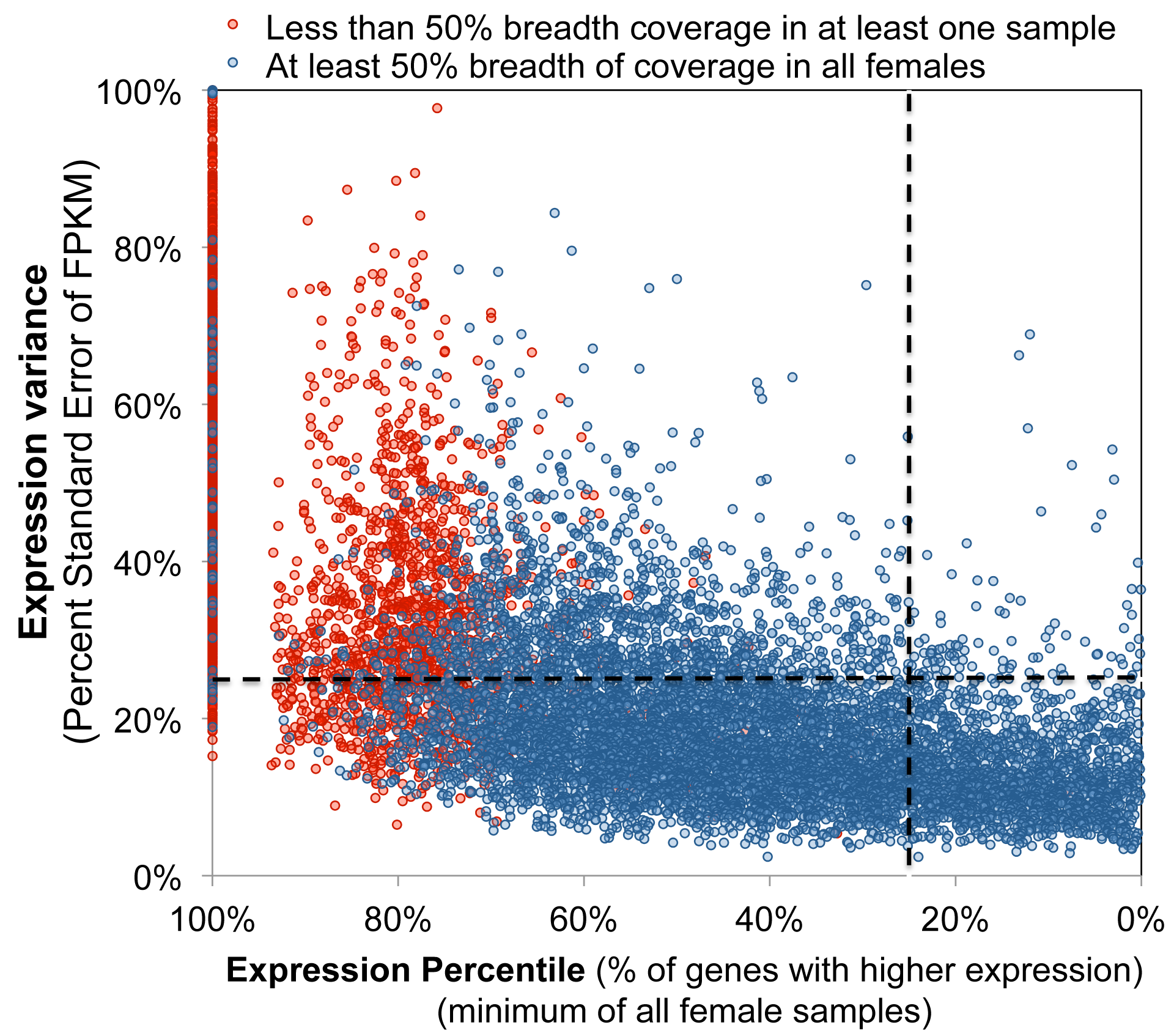
**

**Figure S3: Gene expression-based prioritization of candidate serodiagnostic proteins.**

Genes labeled in blue met the minimum 50% breadth of coverage requirement across all female samples, and blue genes below and to the right of the 25% variance and percentile cutoffs (respectively) were considered to be “consistently” expressed by all three criteria.
