# Supplementary material for: An Integrated Multiomics Approach to Identify Candidate Antigens for Serodiagnosis of Human Onchocerciasis
Source: Mol Cell Proteomics. 2015 Oct 15;14(12):3224–33. doi: 10.1074/mcp.M115.051953 (PMC4762623; doi:10.1074/mcp.M115.051953)
Supplement: Supplemental Data [file supp_M115.051953_Figure_S4.docx]

**Figure S4: Protein sequence alignment of OVOC4612 to the best BLAST matches in three filarial and two hookworm nematode species.**

Accession numbers are as follows: *Necator americanus* ETN81020, *Ancylostomoa ceylanicum* EPB67189, *Loa loa* EFO21093, *Brugia malayi* CDQ04998, *Wuchereria bancrofti* EJW88406.
