# Supplementary material for: An Integrated Multiomics Approach to Identify Candidate Antigens for Serodiagnosis of Human Onchocerciasis
Source: Mol Cell Proteomics. 2015 Oct 15;14(12):3224–33. doi: 10.1074/mcp.M115.051953 (PMC4762623; doi:10.1074/mcp.M115.051953)
Supplement: Supplemental Data [file supp_M115.051953_Figure_S5.docx]

**Figure S5: Protein sequence alignment of OVOC2486 to the best BLAST matches in three filarial nematodes.**

Accession numbers are as follows: *Loa loa* EJD74931, *Brugia malayi* CDP91156, *Wuchereria bancrofti* protein WBA_0000873101-1 from Sanger assembly V2.0.
