# Supplementary material for: An Integrated Multiomics Approach to Identify Candidate Antigens for Serodiagnosis of Human Onchocerciasis
Source: Mol Cell Proteomics. 2015 Oct 15;14(12):3224–33. doi: 10.1074/mcp.M115.051953 (PMC4762623; doi:10.1074/mcp.M115.051953)
Supplement: Supplemental Data [file supp_M115.051953_Figure_S2.docx]

**Figure S2: Clustering of RNAseq samples based on global gene expression patterns.**

Principal component analysis plot of RNAseq samples based on gene expression levels across 500 genes with the highest sample-to-sample variance.
